# Navigating the doctor-patient-AI relationship - a mixed-methods study of physician attitudes toward artificial intelligence in primary care

**DOI:** 10.1186/s12875-024-02282-y

**Published:** 2024-01-27

**Authors:** Matthew R. Allen, Sophie Webb, Ammar Mandvi, Marshall Frieden, Ming Tai-Seale, Gene Kallenberg

**Affiliations:** 1 Department of Family Medicine, University of California San Diego, La Jolla, CA 92093 USA; 2 Division of Biomedical Informatics, University of California San Diego, La Jolla, CA 92093 USA

**Keywords:** Artificial intelligence, Attitudes, Digital work, Primary care, Technology, Qualitative research, Digital health

## Abstract

**Background:**

Artificial intelligence (AI) is a rapidly advancing field that is beginning to enter the practice of medicine. Primary care is a cornerstone of medicine and deals with challenges such as physician shortage and burnout which impact patient care. AI and its application via digital health is increasingly presented as a possible solution. However, there is a scarcity of research focusing on primary care physician (PCP) attitudes toward AI. This study examines PCP views on AI in primary care. We explore its potential impact on topics pertinent to primary care such as the doctor-patient relationship and clinical workflow. By doing so, we aim to inform primary care stakeholders to encourage successful, equitable uptake of future AI tools. Our study is the first to our knowledge to explore PCP attitudes using specific primary care AI use cases rather than discussing AI in medicine in general terms.

**Methods:**

From June to August 2023, we conducted a survey among 47 primary care physicians affiliated with a large academic health system in Southern California. The survey quantified attitudes toward AI in general as well as concerning two specific AI use cases. Additionally, we conducted interviews with 15 survey respondents.

**Results:**

Our findings suggest that PCPs have largely positive views of AI. However, attitudes often hinged on the context of adoption. While some concerns reported by PCPs regarding AI in primary care focused on technology (accuracy, safety, bias), many focused on people-and-process factors (workflow, equity, reimbursement, doctor-patient relationship).

**Conclusion:**

Our study offers nuanced insights into PCP attitudes towards AI in primary care and highlights the need for primary care stakeholder alignment on key issues raised by PCPs. AI initiatives that fail to address both the technological and people-and-process concerns raised by PCPs may struggle to make an impact.

**Supplementary Information:**

The online version contains supplementary material available at 10.1186/s12875-024-02282-y.

## Background

While the potential impact of AI in medicine has been long discussed, real-life, clinician-facing applications of AI have only recently become a reality [1–3]. AI-assisted chronic disease management, diagnostic support, and administrative work (such as documentation, billing, and patient messaging) have significant potential to improve medicine and to take some burden off physicians allowing them to focus on physician-level patient care [4]. Further, use of clinical AI is part of a broader shift in medicine toward “digital health” where many aspects of medical care are conducted remotely, mediated by a technological intermediary leading to potential improvements in efficiency and access [5, 6]. These developments stand to make a substantial impact in primary care, a field that is currently grappling with high rates of physician burnout, inadequate compensation, and a growing shortage of physicians [7–9]. However, there is concern that if AI is poorly integrated it could exacerbate the “disconnect between professional values and the realities of primary care practice” [10, 11]. For example, despite the crucial role of the doctor-patient relationship in medicine, the impact of AI and digital health on this essential component of primary care remains underexplored [12–14]. 

Despite so much on the line, there is limited literature on PCP views toward AI [15, 16]. Much of the research that does exist has taken place in a purely theoretical context exploring AI in general terms with physicians that did not have experience using AI-powered systems. We propose that more end-user engagement with clinicians discussing tangible, specific use cases of clinical AI is needed [17]. By highlighting specific AI use cases, we hope to elicit new concerns and attitudes that would remain hidden when discussing AI in general. Failure to engage end users in the design of AI-powered digital health tools leads to inefficient or unsuccessful integration of these tools into clinical workflow leading to added clinician burnout and even patient harm [10, 18, 19]. 

### Primary care, technology and health equity

Our study recognizes the potential of technology to exacerbate or ameliorate existing inequalities in healthcare [20–24]. AI systems are particularly at risk of worsening health equity due to factors like potentially biased data becoming engrained in AI systems or unequal distribution of newly developed AI tools [25]. Equity considerations are especially vital in the context of primary care. PCPs are often the first point of contact for patients and are central in providing healthcare to communities with limited access due to geographical, economic, or social factors [26–30]. PCPs also make up the largest potential group of AI end users among health professionals [9]. Despite the foundational nature of primary care, this field has long endured a lack of attention, resources, and recognition compared to other medical specialties [31–33]. This has contributed to a comparative lack of AI progress and implementation in primary care in spite of huge need and potential [9, 22, 34–36]. Accordingly, equity is a key consideration for AI in primary care.

### Objective

In our pursuit of user-centric design, we employed a mixed-methods approach to delve into PCP attitudes regarding the potential transformative influence of AI and the broader shift towards digitalization in primary care. Our initial aim is to inform primary care stakeholders of PCP apprehensions regarding potential adverse effects of AI in primary care. Our findings reveal pivotal factors that can either facilitate or hinder the integration of AI systems in primary care. Our long-term aim is to use these findings to develop the AI tools outlined in the manuscript with the goal of improving patient care. While this is not the first exploratory investigation of PCP attitudes about AI, to our knowledge it is the first study that extends beyond the theoretical realm, weaving in specific AI use cases and input from PCPs with real-world experience using primary care AI and digital health tools.

## Methods

### Participant engagement with AI and digital health

Our study participants are affiliated with an academic medical center (AMC) actively engaged in the development, pilot testing, or implementation of several AI applications within its healthcare system. Here, we spotlight specific use cases relevant to primary care, highlighting their pivotal role in the study. These use cases, characterized by their remote and asynchronous elements, also fall under the broader category of digital health [37]. 

### AI-enhanced disease screening: obstructive sleep apnea

Acknowledging the growing prevalence of Obstructive Sleep Apnea (OSA) and its often-undetected status, a research team at our AMC identified OSA as a suitable target for AI-based disease screening [38]. This initiative builds upon prior research employing electronic health record (EHR) data to identify individuals at high risk of OSA [39, 40]. This use case is an archetype for multiple types of disease screening in primary care and raises important questions such as what to do with positive screening results from an AI tool run on a patient panel.

### AI-facilitated disease management: hypertension

Our institution is exploring a digital health strategy for hypertension management, integrating home blood pressure measurements and AI-powered clinical decision support through a panel-level registry [41, 42]. This approach has potential to help primary care physicians give precise hypertension care based on unique patient characteristics while also giving them tools and efficiencies to do so at a population level [42–44]. Additionally, our institution has introduced a population health service enabling PCPs to refer patients with hypertension to digital medication management facilitated remotely by nurses and pharmacists. This use case is an archetype for chronic disease management in primary care and raises questions such as: how can AI augment PCP abilities or coordinate care between different primary care team members?

### AI-facilitated administrative tasks: patient messaging

Inbox overload, which was exacerbated during the Covid-19 pandemic, contributes to burnout and “pajama time” in primary care [45]. To mitigate this challenge, our AMC is currently piloting the utilization of Large Language Models like ChatGPT for drafting patient message responses within the EHR [45]. This use case is an archetype for AI assisting with administrative tasks and raises questions including potential impacts on the doctor-patient relationship.

### Digital survey

As the first step in our mixed-method approach, we employed a digital survey (appendix) specifically developed for our project to quantify PCP attitudes. Given the novel nature of our research focus and the absence of pre-existing validated questionnaires, members of our research team with qualitative research expertise lead the creation of the survey instrument which ensured impartiality, methodological rigor, and ability to capture nuanced insight. Our survey instrument was primarily descriptive in nature and served as a valuable data source for understanding the frequency of responses and providing a framework for the subsequent interviews. Using Likert scales, the survey explored participants’ comfort levels and perceptions about AI in healthcare. We also gathered deidentified demographic data to contextualize perspectives. The survey captured responses from a diverse group of primary care physicians (*N* = 47), providing perspectives from different primary care specialties and practice settings. The sample included physicians from AMC Faculty Internal Medicine (*n* = 6), AMC Faculty Family Medicine (*n* = 36), AMC Clinical Internal Medicine (*n* = 1), AMC Clinical Family Medicine (*n* = 4).

The respondents’ ages encompassed a wide range: 25–34 years old (*n* = 7), 35–44 years old (*n* = 19), 45–54 years old (*n* = 12), 55–64 years old (*n* = 9). Gender diversity was evident with 46.8% male (*n* = 22) and 53.2% female (*n* = 25) respondents. Years of experience in practice varied (mean = 3.17 years; *n* = 47), including 1–5 years (*n* = 15), 6–10 years (*n* = 6), 11–15 years (*n* = 8), 16–20 years (*n* = 5), 21–25 years (*n* = 5), 26–30 years (*n* = 4), 31–35 years (*n* = 3). This spectrum ensures broad insights into PCP perspectives across different demographics and career stages.

### Semi-structured interview

In the survey, respondents were asked if they would be willing to engage in a follow-up interview. Employing a semi-structured interview format with an interview guide (appendix) iteratively developed for our project in conjunction with qualitative research experts, we provided participants with an open and adaptable platform to share their perspectives which we then scrutinized using thematic analysis. Interviews were conducted in a confidential environment via remote teleconferencing software (Zoom). Automated transcription software (Otter.AI) was used for transcription generation and collected interview data underwent rigorous thematic analysis using Quirkos qualitative analysis software. This method involved a systematic process of coding and categorizing responses to identify recurring patterns, insights, and emerging themes. Through iterative refinement, we extracted meaningful themes that captured the essence of PCP’s views regarding AI in primary care. Following our initial interviews (*n* = 6) that highlighted PCP’s concerns about increased workload and expectations, we added a question to delve deeper into how AI could affect the doctor-patient relationship. Our final number of interviews was 15.

## Results

### General perceptions of a.i. in medicine

The majority of survey respondents (76.6%) held an optimistic perspective regarding the potential of AI in medicine. Comfort levels in integrating AI-based technologies into clinical practice varied across different domains (Table 1).

**Table 1:** Table percentages represent the proportion of PCPs reporting varying levels of comfort with AI involvement in different domains as reported via the digital survey

Domain	Very Comfortable	Somewhat Comfortable	Neutral	Somewhat Uncomfortable	Very Uncomfortable
Disease Screening	29.8%	46.8%	8.5%	8.5%	6.4%
Chronic Disease Management	25.5%	46.8%	12.8%	8.5%	6.4%
Disease Diagnosis	8.5%	42.6%	10.6%	23.4%	14.9%
Administrative Tasks	40.4%	25.5%	12.8%	14.9%	6.4%

While some physicians reported feeling comfortable communicating the role of AI-based tools to patients (very comfortable: 6.4%, somewhat comfortable: 36.2%) a sizeable percentage did not (somewhat uncomfortable: 23.4%, very uncomfortable: 12.8%). Importantly, 70.2% of surveyed physicians described their approach to learning about AI in medicine as “passively learning via popular news sources or casual conversation” with only 25.5% “actively seeking education through established organizations, coursework, lectures, professional journals, or books.”

### Concerns about AI in primary care

**Fig. 1:**
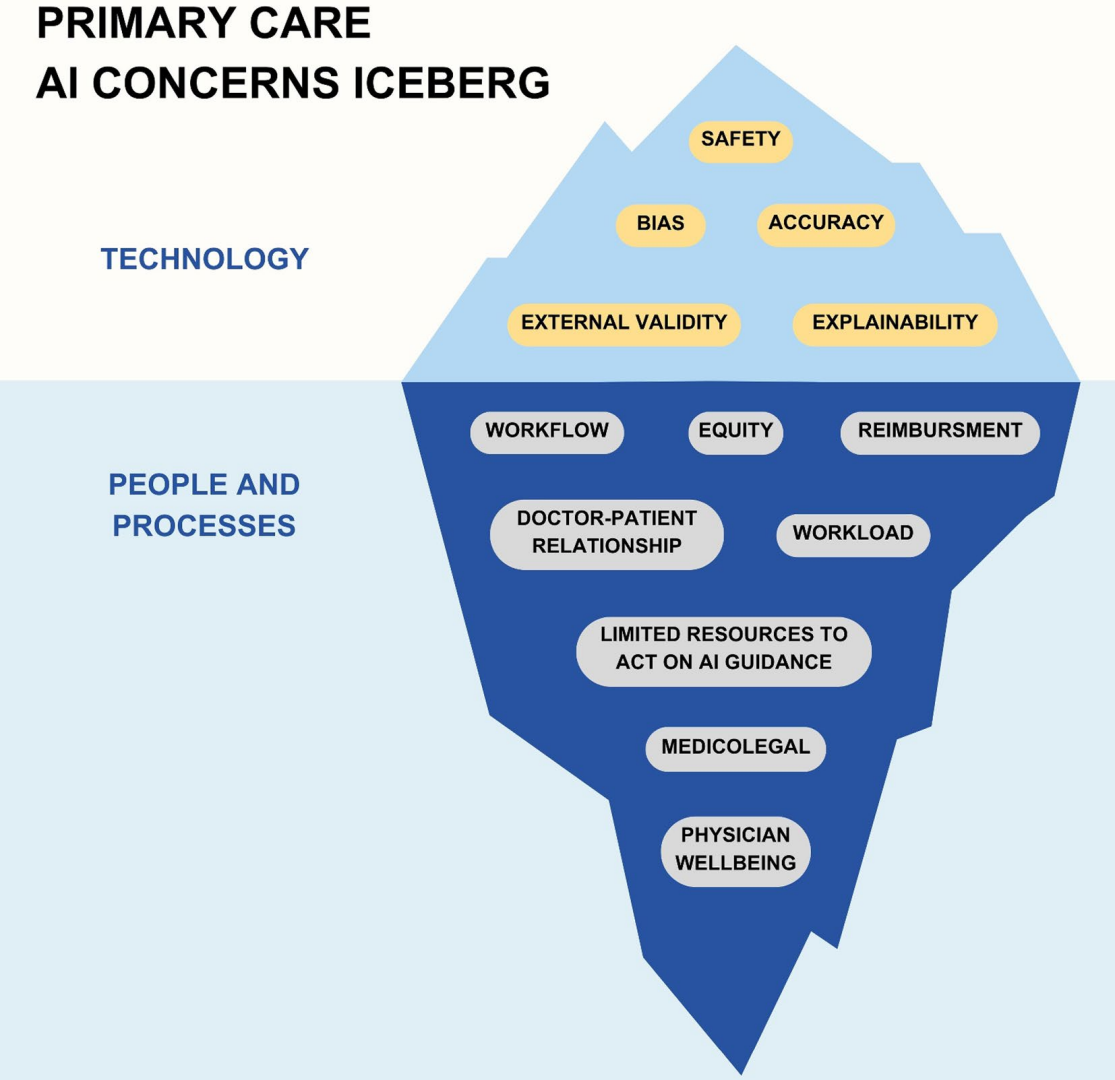
PCP concerns about AI. Description: Emergent themes from interviews with primary care physicians regarding AI divided into concerns about AI technology itself and concerns about the context and manner of AI implementation

Despite the general positivity quantified in the survey, interview participants expressed numerous concerns about AI—especially when discussing specific AI use cases. We subjected our interview data on concerns regarding AI to a thematic coding analysis and identified the following themes which have been categorized as concerns regarding technology or people-and-processes (Fig. 1) [46]. 

Technological concerns included factors such as algorithmic bias (1 participant) or accuracy and safety (7 participants).

*The thing I’m apprehensive about is, how are we teaching AI these things because some of those biases could leak in.* [Participant C]

*My concerns around AI in medicine have most to do with the space of accuracy. And a tool that I feel is reliable.* [Participant G]

Concern about external validity and the ability of AI algorithms to appreciate the nuances of specific patients (8 participants) was an important consideration for PCPs.

*I’ve known a lot of my patients now for 30 years and know a lot about them. That can’t really be put into a data set that AI can draw upon.* [Participant F]

Interviewed PCPs reported differing opinions on whether they prioritized explainability [47] in AI models (3 participants),

*At this point, I want to be able to get a logical explanation.* [Participant L]

or if this was not imperative for them (10 participants).

*I’m perfectly okay if my own experience with time goes better and better and I feel like, you know, it works. Don’t ask me how it works, but it works.* [Participant A]

*I almost think that the tool adds more to the decision-making process if it’s operating outside of that human accessible reasoning process.* [Participant B]

However, many reported concerns centered around systemic issues rather than technological ones. One common concern (5 participants) was that of the medicolegal implications of acting, or failing to act, on AI guidance.

*If the system is saying, ‘Hey, this person has severe sleep apnea’, and what if they get in a car accident tomorrow and we had that data today?* [Participant O]

When discussing the potential of algorithmic detection of OSA, PCPs (5 participants) pointed out that without augmenting the system’s ability to definitively diagnose and treat more patients with OSA, the AI tool would not be helpful.

*When we are loading our system from this side, we need to have the resources on the other side.* [Participant E]

*I think we need to be better equipped before we start telling people this because, you know, it’s like, Hey, you might have sleep apnea. Wait six months for your sleep study…* [Participant C]

Additionally, 10 participants reported concern that the integration of AI into primary care could potentially lead to increased workload and physician burnout.

*My concern is that like everything else that we have tried to do to make things better in medicine is that it actually makes things harder on the physician and creates more work for us instead of less work.* [Participant D]

Interviewees reported multiple ways in which this could happen including AI tools delegating work to physicians that could potentially be handled by other team members,

*Is it really the physicians that should deal with this in the first place?* [Participant K]

a need to constantly verify or redo work done by AI,

*It’s like having a student with me all the time, where I’ve got to just double check everything.* [Participant D]

or an excessive focus on productivity.

*We are going to add two extra patients per session because now we have help there. So unfortunately, sometimes more help is used in a negative way.* [Participant E]

*I’ve always seen that the system wants productivity, and the way productivity is defined is based on the number of patients seen.* [Participant L]

Not all PCPs shared this concern of increased workload due to AI, with one participant expressing that increased efficiency due to AI would be welcome even if it meant seeing more patients.

*If I could see 30 patients in a day, and actually close out my charts by 6pm, smiling, and get home for dinner, I’d be happy.* [Participant G]

Physicians also reported concerns on how AI might impact the doctor-patient relationship. Some expressed positive hopes for AI to improve the doctor-patient relationship (10 participants) by doing things such as alleviating clinician burden or improving patient engagement.

*Maybe you are actually then more compassionate in an encounter, because you haven’t had to do all of that mental lifting.* [Participant M]

But many—some that had also expressed positive sentiment—worried that AI could harm the doctor-patient relationship (12 participants) by factors such as warping patient expectations or prioritizing patient needs over physician well-being.

*Patients may end up feeling that, you know, if the AI can tell me that then why did I bother to come to you?* [Participant L]

*The more we sort of train patients to expect things quickly and efficiently, the more expectations are on the doctor to then produce in the same way.* [Participant D]

Participating PCPs also lamented a lack of focus on physician-wellbeing when implementing new technologies (9 participants).

*I feel like right when I get efficient, something new gets introduced.* [Participant M]

*The system is all about the patient’s satisfaction. Is there any of that focus on physician satisfaction?* [Participant E]

One key point was the concern that the current way healthcare is paid for does not encourage innovative ways of care delivery such as AI-powered digital health tools.

*In essence, we’re providing a bunch of free care, which, you know, is not sustainable.* [Participant H]

Thus, it appears that PCPs see a disconnect between care innovation and the way they are forced to practice due to how care is reimbursed.

*They’re told to do both things. So they’re really there to crank it out, crank out these RVUs while also doing value based medicine and population based medicine.* [Participant J]

Dedicated time for digital health as well as alternative reimbursement models were frequently voiced (11 participants) as a key determiner of the uptake and success of AI tools.

*I’d like dedicated time daily or at least weekly to review. Otherwise, I might only see it if I see the patient.* [Participant F]

*I think that this system really needs to rethink how it employs physicians and providers.* [Participant L]

Data from our survey corroborates this finding that PCPs are unsure of where digital health tools fit into their workflow. When asked about preferences about when to receive communication from an AI tool regarding a patient screening positive for OSA, responses varied widely with 3 respondents using the free response to indicate that they would *not* like to be notified. When asked if they were aware of a pre-existing EHR registry of patients with hypertension, nearly half (45.83%) responded no. For those that were aware of the registry, more than half reported that their usage of the registry was “infrequently” (30.77%) or “never” (23.08%) with the most common reported reason being a lack of time.

## Discussion

### AI as a double-edged sword

Our findings reveal the dual nature of AI in healthcare, uncovering its potential to alleviate or exacerbate challenges in primary care. Some of our identified concerns about AI adoption in healthcare, including lack of external validity, potential for bias, and safety issues, have been well-documented in the literature [48–50]. Our study expands upon these concerns highlighting that, for clinicians, the mechanics of AI itself may take a back seat to its potential impact on their professional lives, personal well-being, and their relationships with patients [51]. We argue that concerns such as apprehension about increased workload stem from a broader sentiment among PCPs that advancements in healthcare often prioritize productivity over physician well-being or put financial considerations over human relationships [52–54]. Accordingly, some PCP concerns about AI may be a reflection of a disillusionment with the evolving landscape of medicine in general. In this context, the introduction of AI is perceived as yet another instance where physician interests may be subjugated to organizational efficiency. These concerns are not unfounded with previous literature proposing using technology to add capacity as one of the solutions to keep up with the increasing physician shortage [55, 56]. Further, PCP panel sizes are already felt to be excessive and a fear regarding AI being used to justify the addition of patients may be rational [57–59]. Digital health and AI in primary care must be applied thoughtfully to avoid further ostracizing PCPs from their professional values.

*I think we all worry that more work is what things are aimed at.* [Participant F]

These are not selfish concerns as the well-being of physicians is intrinsically linked to patient outcomes and is aligned with the Quadruple Aim of healthcare [60, 61]. This concern for physician well-being is especially pertinent in the context of primary care—a cornerstone of healthcare critical for providing access to underserved populations that is chronically undervalued by the healthcare system [62, 63]. PCPs should be able to share in benefits such as time or cost-savings produced by the implementation of AI systems. Our findings suggest that if all benefit goes to the organization, physician appetite for uptake will remain low.

### Navigating the doctor-patient-AI relationship

**Fig. 2:**
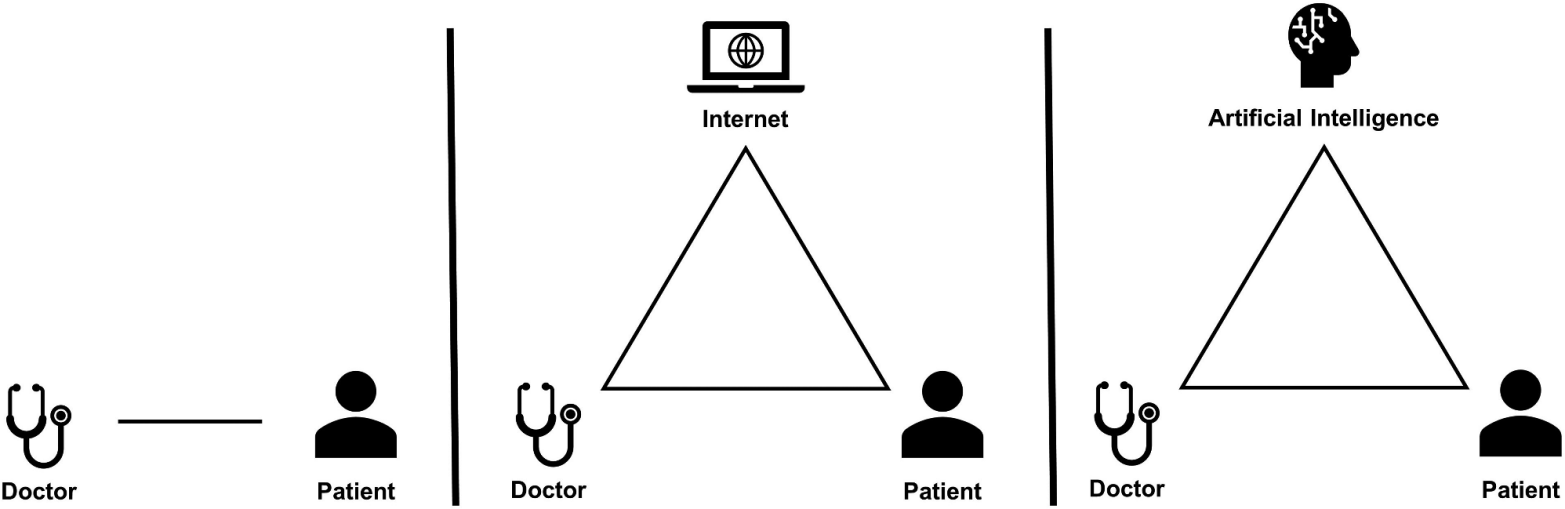
The evolution of the doctor-patient relationship. Description: The advent of the internet had significant impacts on the doctor-patient relationship. Primary care physicians have a mix of concern and optimism about how AI may do the same

Our work also highlights the evolving role of the primary care physician [64]. Once commonly viewed as the source of medical truth, physicians now coexist with “Dr. Google,” a digital repository of health information that empowers patients to engage proactively in their own care [65]. This change has had mixed effects on the doctor-patient relationship but can be positive if both parties engage in proper communication and shared-decision making [66–68]. These positive effects also hinge on factors such as strong patient health information literacy and adequate doctor-patient communication time [69]. Against this backdrop, The impending integration of AI into healthcare will similarly revolutionize the doctor-patient dynamic (Fig. 2).

Much of the same research that was done to explore the impact of internet health information on the doctor-patient relationship needs to be repeated and expanded upon in the context of AI. Unlike static online information, interactive AI systems are poised to assume a more active role in shaping the interactions between patients and physicians. Ensuring a positive impact of AI on the doctor-patient relationship is essential to maintaining medicine’s social contract with society [70]. 

AI’s role in providing patients with information, be it accurate or misleading, could confound the physician-patient dynamic. Patients arriving with AI-informed information could complicate collaborative decision-making [71]. Possible consequences include rigid adherence to AI-driven advice without considering individual medical history or difficulties for physicians attempting to reconcile their expertise with AI suggestions. This not only risks eroding the PCP’s role but also could reshape the doctor-patient relationship into a consumer-provider model.

*I’m worried about AI, introducing a dynamic where misinformation is enhanced… if a patient comes in and they’re like, hey, like, you know, WebMD.GPT told me that I need an MRI then there’s another powerful thing that I’m arguing against.* [Participant B]

A key part of ensuring a positive impact of AI on the doctor-patient relationship is promoting realistic and aligned expectations regarding AI via education for doctors and patients before implementation of AI tools.

*Instead of just letting the cat out of the bag and seeing what happens, you want to make sure that everyone that is going to be interacting with it has accurate expectations and has been educated on what role this is supposed to play.* [Participant J]

Previous literature has highlighted concerns that unequal knowledge or differing backgrounds in the doctor-patient relationship could exacerbate health inequity [72, 73]. Thoughtful and equitable implementation of AI could encourage increased patient engagement and understanding leading to more effective doctor-patient communication and increased equity.

### The future of primary care workflow

Our findings call for a reconsideration of fundamental questions regarding primary care workflow. If PCPs are going to be active participants in new forms of healthcare delivery, including AI-powered digital health, when are they supposed to *do* that work? This shift toward digital health is already occurring albeit in an unscheduled and uncompensated way [45, 74, 75].

*“Most of our care is delivered in MyChart. Like let’s just be honest, that’s how it’s getting delivered. *[Participant G]

Inbox burden is a well-known problem, but it is only the beginning of asynchronous digital versus synchronous in-person workload conflict in primary care [76]. The failure of health systems to identify proper ways of allocating time, resources, and standards to these new ways of interacting with patients has already had substantial consequences [77]. AI-powered digital tools for chronic disease management and disease screening augmented by remote patient monitoring systems will likely become increasingly common [78, 79]. Accordingly, consideration needs to be given to how best to allocate physician time to support digital health. Succeeding in digital health is more than solving inbox overload or alarm fatigue but rather realizing a fundamental shift in how primary care interacts with and takes care of patients [6]. We propose that experimenting with hybrid in-person and virtual work schedules could empower physicians to actualize the potential of digital health [80]. 

Another consideration is: how can AI be integrated into patient-centered, team-based primary care [81]? A population health approach to primary care consists of a physician acting as a “healthcare quarterback” who is responsible for the health of an entire patient panel, regardless of how, where, or by whom each component of care is delivered [81, 82]. Our interview participants frequently indicated that physicians need not always be the primary point of contact for an AI recommendation. Identifying when other team members can review and act upon AI-produced guidance while maintaining the PCP in the loop could mitigate concerns around the possibility of AI and digital health creating more work for physicians [82, 83]. This must be done carefully in a manner that enhances—rather than erodes—the core doctor-patient relationship [11, 12]. 

**Fig. 3:**
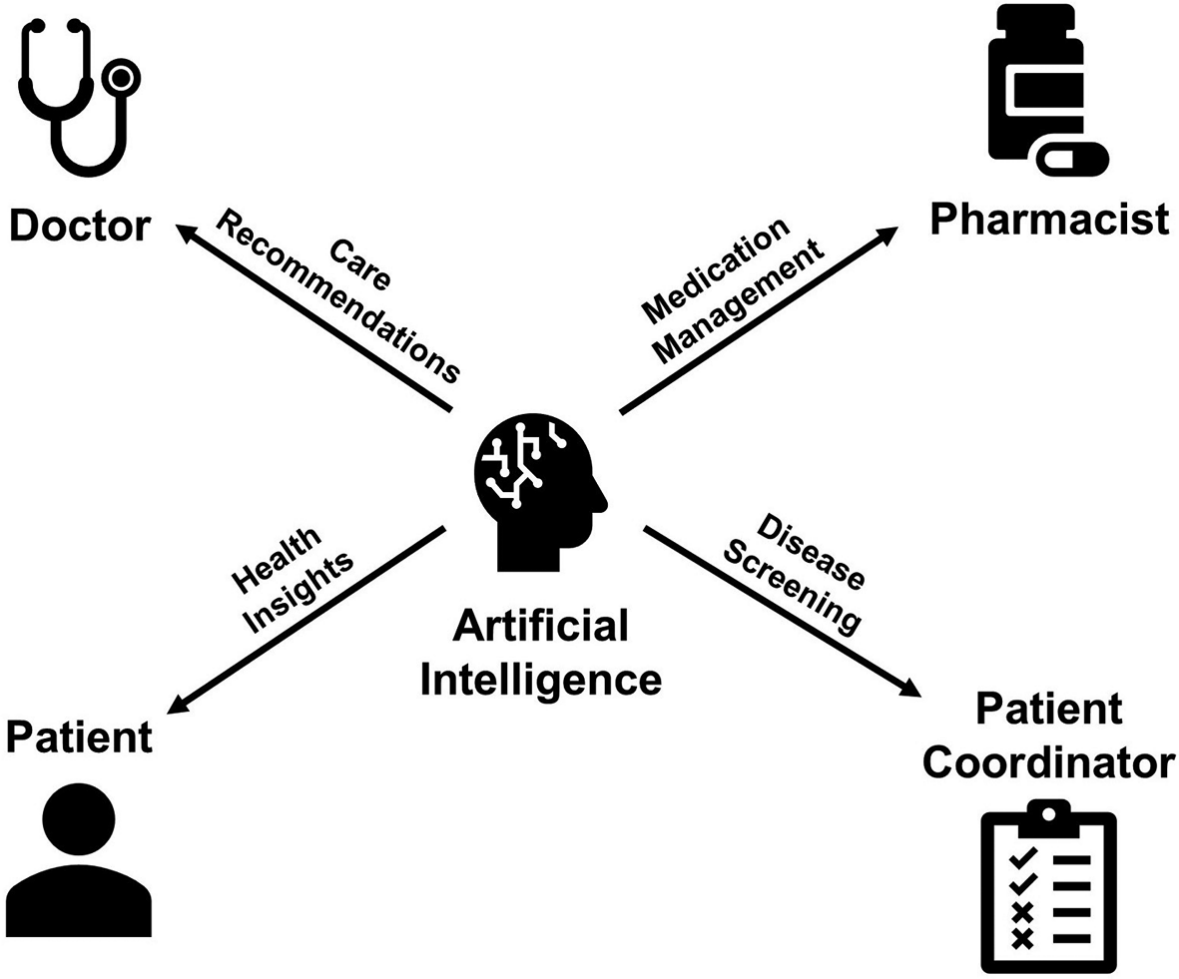
AI as a member of the healthcare team. Description: AI becoming a member of the primary care team

In a more general sense, care coordination is a core challenge of primary care. More research should be done on using AI as a facilitator of task follow-up, delegation, and other components of care coordination (Fig. 3) [84]. Designing standardized primary care workflow is challenging [85]. However, enabling PCPs with time, team members, and incentives to use AI-powered digital tools that handle some aspects of care remotely and asynchronously could facilitate more meaningful, effective, and focused in-person clinic visits [86–88]. More time spent on digital avenues of care has also been shown to improve quality of care metrics [89]. Currently, PCPs already engage in this sort of work but they do it at the expense of time with their patients or their families [75, 90]. Organizations should be wary of relying too much on physician altruism to find time to use digital tools, forcing physicians to choose between their personal wellbeing and that of their patients [91–93]. 

### Considerations for primary care stakeholders

As a first step, stakeholders need to ensure that primary care AI systems are rigorously evaluated from an accuracy, safety, and bias standpoint. However, subsequent attention must be given to workflow integration and impact on physician well-being (Table 2) [86, 94]. Previous literature has shown that such non-technical factors are essential to promote uptake of new technologies [95]. Caution must be taken to ensure that AI does not result in “less doctor work and more office work” leading to PCPs who feel exploited by the healthcare system [10]. Rather, AI should be used as an opportunity to address primary care challenges such as helping deliver better care while preventing physician burnout [96]. 

Additionally, the way in which healthcare is compensated has a substantial impact on the behavior and time-allocation of PCPs [97]. Some attempts, such as charging a user fee for patient messages and billing payers for e-visits (i.e., responding to patient messages), have been made to reimburse digital health services in a fee-for-service model [98]. However, digital health is likely more well suited for value-based primary care that would incentivize and provide flexibility for physicians to engage in asynchronous, population-level digital health tools [99]. Organizations wishing to reap the benefits of AI in primary care must tackle this challenge head on and be willing to reimagine how care is delivered and paid for rather than further ingraining legacy systems and approaches. This is especially pertinent in considering the nature of healthcare reimbursement in the United States. Efforts at payment modernization are underway, [100] but failure to quickly advance and innovate our payment models could lead to our systems lagging behind non-fee-for-service nations in terms of AI and digital health innovation.

**Table 2:** Recommendations for primary care stakeholders

Stakeholder	Recommendation
Payors	Implement innovative reimbursement models for PCPs and other primary care team members engaging in digital health
Healthcare Systems	Schedule time and establish standards for PCPs to engage in digital health
Healthcare Systems	Provide PCPs with additional team members such as pharmacists or patient coordinators who can engage digitally with patients
Healthcare Systems	Develop and disseminate educational materials on the proper role of new AI tools to patients and physicians before tool implementation
Researchers	Run RCTs, pragmatic trials, or practice-based research between traditional and digitally enhanced PCP workflows
Researchers	Evaluate new AI-powered digital tools in the context of physician workflow instead of an isolated environment
PCPs	Advocate individually and collectively for AI tools that improve physician care quality, well-being, and the doctor-patient relationship

### AI and equity

The people who will get it are the people who can pay for the compute. And so that’s my biggest fear is that we will leave out the poorest people from getting the best care. – Participant G.

This sentiment expressed by multiple study participants underscores the gravity of ensuring equitable access to AI-powered healthcare solutions for patients across the spectrum of socioeconomic backgrounds. The transformation brought by AI should not inadvertently reinforce existing disparities, but rather serve as a tool to alleviate them [101]. For example, AI must not alleviate physician burnout and improve patient outcomes only at large AMCs that have adequate resources to engage in AI development and implementation but should be broadly accessible and applicable across diverse healthcare settings and institutions, ensuring equitable access to its benefits for all. Safety-net health systems, federally qualified health centers, rural areas, and other practice settings that could be left out need to be included in the primary care AI revolution [102–104].

The reality is that our patients have much more healthcare that they need delivered than we can ever deliver, so they’re going to need AI tools. – Participant G.

### Strengths, limitations, and directions for future research

In contrast to much of the existing literature, all our study participants have had actual experience with digital health and some of our study participants have actual experience with medical AI. Because these technological shifts are just beginning, clinicians with perspectives informed by actual experience are rare, making our findings more valuable. However, a limitation is that terms such as “AI” or “digital health” have evolving definitions and may not mean the same thing to different individuals. While we tried to ameliorate this effect by grounding our discussions in tangible use-cases and examples, participants differing preconceived ideas of AI may have affected participant responses. Further, focusing on certain use-cases over others may have influenced reported PCP views on AI in general. Future efforts should more comprehensively evaluate perceptions of AI in primary care to ensure that reported PCP attitudes are not overly influenced by any particular use-case. Additionally, while our survey and interview guides were developed in a rigorous manner in collaboration with qualitative methods experts, future research should attempt to develop validated qualitative tools for assessing PCP attitudes toward AI in primary care.

Our research focused only on internal medicine and family medicine physician attitudes toward AI and digital health. Future research needs to include other primary care team members including primary care pediatricians, nurses, and advanced practice providers such as nurse practitioners and physician assistants. In addition, greater research is needed on how patients—especially those that may be marginalized—are experiencing a shift toward AI and digital health in primary care [105, 106]. Finally, future research should also expand beyond our selected AI use cases to incorporate other AI applications pertinent to primary care.

Our relatively small sample size limits the generalizability of our findings to larger populations. Additionally, all our respondents originated from the same organization. While we attempted to assess attitudes of both academic and non-academic physicians, this organizational homogeneity might further limit generalizability. Moreover, the potential for response bias in self-reported responses should be acknowledged.

In light of these limitations, we emphasize the need for future research endeavors to employ quantitative methods to explore questions regarding AI in primary care and to incorporate larger and more diverse samples from various healthcare settings. Incorporating multiple organizations—especially those that are not well-funded AMCs in urban environments—can provide a broader perspective on the adoption of AI and digital health in primary care.

## Conclusion

This study was the first to investigate PCP attitudes toward AI in primary care focusing on specific AI use cases. Reported attitudes varied, but PCP responses showed general optimism around AI in primary care tempered by certain concerns. While some concerns focused on technological factors like algorithmic accuracy, safety, and bias, others focused on people-and-process factors such as effects on physician workflow, equity, reimbursement, and the doctor-patient relationship. These findings suggest that AI initiatives that fail to address both the technological and people-and-process concerns raised by PCPs may struggle to make an impact. Primary care stakeholders should use these findings to inform development and implementation of AI in primary care.

### Electronic supplementary material

Below is the link to the electronic supplementary material.

**Supplementary Material 1:** Question stems from the digital survey

**Supplementary Material 2:** Questions stems from the semi-structured interview

## Data Availability

The digital survey questions and interview guide question stems will be included as an appendix to the manuscript. Complete interview and survey data will be made available upon reasonable request.

## Abbreviations

AI: Artificial Intelligence
PCP: Primary Care Physician
AMC: Academic Medical Center
OSA: Obstructive Sleep Apnea
EHR: Electronic Health Record
