# Supplementary material for: Navigating the doctor-patient-AI relationship - a mixed-methods study of physician attitudes toward artificial intelligence in primary care
Source: BMC Prim Care. 2024 Jan 27;25:42. doi: 10.1186/s12875-024-02282-y (PMC10821550; doi:10.1186/s12875-024-02282-y)
Supplement: Supplementary file 1 — Supplementary Material 1: Question stems from the digital survey [file 12875_2024_2282_MOESM1_ESM.docx]

**Semi-Structured Interview Guide**

**Consent Statement**

“This is a follow-up interview to the digital survey you completed regarding AI in primary care. You are under no obligation to participate, to answer every question, or to continue at any point in the study. Your answers will be anonymized in any publications. We would also like to record this interview to prepare a transcript for subsequent analysis.

Do you give your oral consent to participate?”

*If yes, the interview proceeded.*

**Part One – General Discussion of AI in Primary Care**

- What are your main concerns regarding use of AI in medicine?
- How do you feel that AI will change the doctor patient relationship?
  - *(Added after interview #6)*
- What characteristics would make you trust or distrust an AI system?

**Part Two – Discussion of Specific AI Use-Cases**

*Participants were given a brief recap of the basic design characteristics of each tool that was presented to them initially in the digital survey (Population Hypertension Management and Obstructive Sleep Apnea Prediction).*

- Do you have any initial feedback on the tool we are developing?
- How would this tool best fit into your workflow?
- What barriers do you foresee in implementing this tool?
